# Correction to: American clusters: using machine learning to understand health and health care disparities in the United States

**DOI:** 10.1093/haschl/qxae146

**Published:** 2024-12-09

This is a correction to: Diana M Bowser, Kaili Mauricio, Brielle A Ruscitti, William H Crown, American clusters: using machine learning to understand health and health care disparities in the United States, *Health Affairs Scholar*, Volume 2, Issue 3, March 2024, qxae017, https://doi.org/10.1093/haschl/qxae017

In the originally published version of this manuscript, there was a typographic error in author Kaili Mauricio's name. This error has been corrected.

